# Disulfiram Transcends ALDH Inhibitory Activity When Targeting Ovarian Cancer Tumor-Initiating Cells

**DOI:** 10.3389/fonc.2022.762820

**Published:** 2022-03-17

**Authors:** Michael W. Caminear, Brittney S. Harrington, Rahul D. Kamdar, Michael J. Kruhlak, Christina M. Annunziata

**Affiliations:** ^1^ Women’s Malignancies Branch, National Cancer Institute, National Institutes of Health, Bethesda, MD, United States; ^2^ Center for Cancer Research (CCR) Confocal Microscopy Core Facility, Laboratory of Cancer Biology and Genetics, Center for Cancer Research, National Cancer Institute (NIH), Bethesda, MD, United States

**Keywords:** ALDH1A1, ALDH inhibitors, disulfiram, ovarian cancer, tumor-initiating cells

## Abstract

Epithelial ovarian cancer (EOC) is a global health burden and remains the fifth leading cause of cancer related death in women worldwide with the poorest five-year survival rate of the gynecological malignancies. EOC recurrence is considered to be driven by the survival of chemoresistant, stem-like tumor-initiating cells (TICs). We previously showed that disulfiram, an ALDH inhibitor, effectively targeted TICs compared to adherent EOC cells in terms of viability, spheroid formation, oxidative stress and also prevented relapse in an *in vivo* model of EOC. In this study we sought to determine whether specific targeting of ALDH isoenzyme ALDH1A1 would provide similar benefit to broader pathway inhibition by disulfiram. NCT-505 and NCT-506 are isoenzyme-specific ALDH1A1 inhibitors whose activity was compared to the effects of disulfiram. Following treatment with both the NCTs and disulfiram, the viability of TICs versus adherent cells, sphere formation, and cell death in our *in vitro* relapse model were measured and compared in EOC cell lines. We found that disulfiram decreased the viability of TICs significantly more effectively versus adherent cells, while no consistent trend was observed when the cells were treated with the NCTs. Disulfiram also affected the expression of proteins associated with NFκB signaling. Comparison of disulfiram to the direct targeting of ALDH1A1 with the NCTs suggests that the broader cellular effects of disulfiram are more suitable as a therapeutic to eradicate TICs from tumors and prevent EOC relapse. In addition to providing insight into a fitting treatment for TICs, the comparison of disulfiram to NCT-505 and -506 has increased our understanding of the mechanism of action of disulfiram. Further elucidation of the mechanism of disulfiram has the potential to reveal additional targets to treat EOC TICs and prevent disease recurrence.

## Introduction

The stem-like tumor-initiating cell (TIC) populations in epithelial ovarian cancer (EOC) are considered the main drivers of relapse in EOC. The TIC population is characterized by enhanced chemoresistance, resistance to cell death, oxidative stress mitigation, and the ability to reestablish cancer after the bulk population is reduced by first line, broad-targeting treatments (1–4). TIC populations in EOC can be identified by the expression of molecular markers, namely, CD133, *NANOG*, and *SOX2* expression (5). Despite extensive characterization of identifying markers of TICs, these markers provide little opportunity for therapeutic targeting. However, high aldehyde dehydrogenase (ALDH) activity is a marker of EOC TICs and is also functionally important, promoting therapy resistance, cancer stem cell maintenance and oxidative stress mitigation and is being investigated as a therapeutic target (6, 7). ALDH is associated with poor prognosis in several cancers, namely, breast, prostate, lung, and ovarian cancer (6, 8–10). In ovarian TICs, the methods by which ALDH maintains TIC survival include the robust catalyzation of aldehyde oxidation conferring drug resistance and the activation of nrf2 signaling to manage high levels of oxidative stress (11–13). The reported high levels of ALDH activity in EOC make it an attractive therapeutic target for inhibition (14).

We recently investigated differences in drug sensitivities between EOC cells grown in TIC-enriching spheroid and adherent growth conditions to identify potential therapeutics for targeting TICs (15). We found that TICs were sensitive to the broad ALDH inhibitor disulfiram compared to adherent cells, and that disulfiram delivered intraperitoneally could prevent relapse in a post-surgery, post-chemotherapy ovarian cancer mouse model (15). While disulfiram is an approved drug used historically in the treatment of alcoholism and provides broad ALDH inhibition, it is not formulated for systemic delivery and is rapidly metabolized by the liver, its primary target organ (16). For this reason, we sought alternative and specific compounds to target the ALDH isoenzyme that was overrepresented in EOC TICs, ALDH1A1 (11, 17).

There are a number of ALDH1A1 isoform-specific and multi-isoform targeting inhibitors being investigated as anti-cancer agents (18, 19). In this study, we aimed to compare the inhibitory activity of specific isoenzyme inhibitors to the broader targeting disulfiram, to determine the effectiveness of targeting ALDH1A1 in treating TICs. We investigated the specific ALDH1A1 inhibitors, NCT-505 and NCT-506, that were previously developed and tested on ovarian cancer cell lines and were shown to sensitize paclitaxel-resistant ovarian cancer cells (20).

## Results

### ALDH1A1 Expression and ALDH Activity in Ovarian Cancer Spheroids

**Figure 1:**
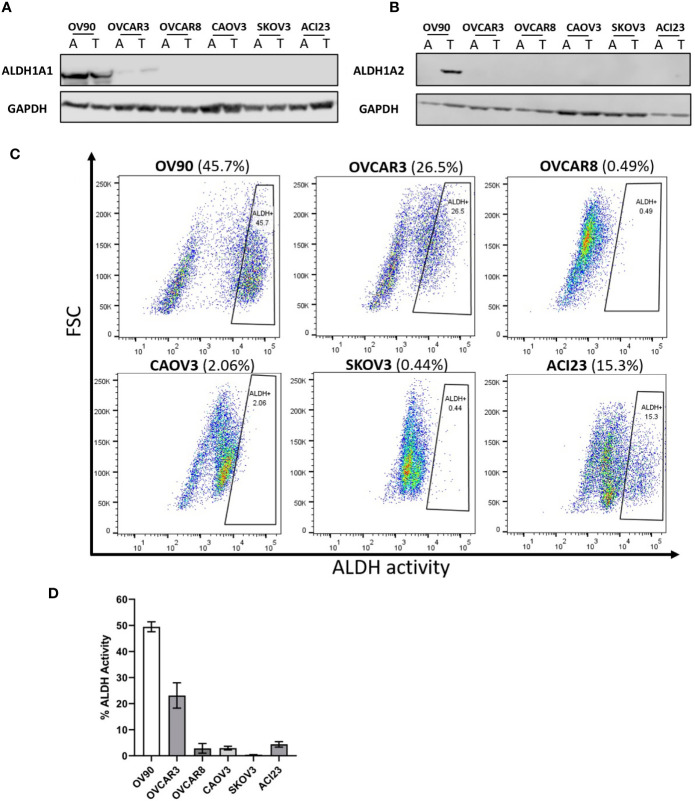
Detection of ALDH1 isoforms in ovarian cancer cell lines by Western blot analysis, and measurement of ALDH activity in TICs determined by ALDEFLUOR flow cytometry assay. Western blot analysis of lysates probed for the expression of **(A)** ALDH1A1 and **(B)** ALDH1A2 in the indicated cell lines in adherent and TIC growth conditions. **(C)** The ALDH-positive population of TICs in the indicated cells lines detected *via* flow cytometry after 72 h in TIC growth conditions. **(D)** Quantified percentages of ALDH activity in cell lines (n = 2). Graphs represent mean and SEM of each indicated cell line. A, adherent cells; T, TICs.

Previously, we and others found that ALDH1A1 and ALDH1A2 mRNAs were upregulated in TIC growth conditions (5) and that ALDH activity is increased in TIC-enriching spheroid growth conditions under the influence of RelB transcriptional activity (17). We selected EOC cell lines with high ALDH activity to test the ALDH1A1 specific inhibitors, NCT-505 and NCT-506. Western blot analysis of EOC cell lines showed that ALDH1A1 protein expression was detected in OV90 and OVCAR3 cell lines, in both adherent and TIC-enriching (spheroid) conditions, but was not detected in OVCAR8, CAOV3, SKOV3 or ACI23 lines (**Figure 1A**). This is consistent with previous reports (20). ALDH1A2 expression was detected only in OV90 cells grown in spheroid conditions (**Figure 1B**). ALDH activity was measured in EOC cells grown in spheroid conditions, and OV90 and OVCAR3 had the highest amount of ALDH activity, followed by ACI23, OVCAR8, and CAOV3 (**Figures 1C, D**). Based on these expression data, we investigated the effects of the specific ALDH1A1 inhibitors on OV90 and OVCAR3 cells. The cell line OVCAR8 was used as a comparator, since its ALDH activity and expression was relatively low, but we previously showed this line was sensitive to the broad ALDH inhibitor disulfiram, suggesting effects beyond ALDH inhibition (15).

### Significant Reduction of ALDH Activity in TICs Treated With Disulfiram, NCT-505, and NCT-506

**Figure 2:**
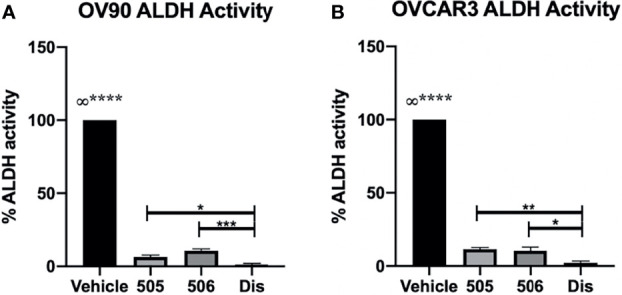
ALDH inhibitors significantly reduced ALDH activity; disulfiram reduced activity significantly more than NCT-505 and -506. ALDH activity after treatment with calculated viability IC_50_ of NCT-505, NCT-506, and disulfiram determined in **(A)** OV90 and **(B)** OVCAR3 TICs *via* flow cytometry following ALDEFLUOR protocol. Graphs represent mean and SEM of each treatment, from 3 independent experiments, ∞, compared to all groups, *p <0.05, **p < 0.01, ***p < 0.001, ****p < 0.0001, compared to vehicle control.

The specific inhibitors were compared to disulfiram for their ability to inhibit ALDH activity in OV90 and OVCAR3 spheroids as these lines had the highest ALDH activity of the EOC cells tested. The spheroids were treated with disulfiram or the NCT-505 or -506 compounds for 72 h at the calculated viability IC_50_ doses for each drug and cell line and activity was assessed by the ALDEFLUOR assay (5, 15, 17). All drugs significantly diminished ALDH activity in spheroids compared to vehicle control but disulfiram did reduce ALDH activity to a greater level than NCT-505 and -506 at the selected concentrations (**Figures 2A, B**).

### Specific ALDH1A1 Inhibitors NCT-505 and NCT-506 do not Have a Selective Effect on Spheroid Viability, Compared to Disulfiram

**Figure 3:**
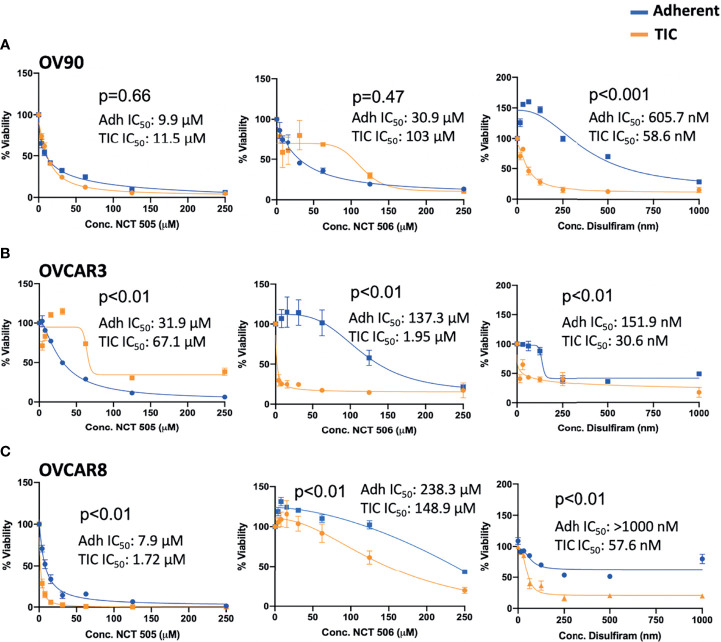
Viability of **(A)** OV90, **(B)** OVCAR3, and **(C)** OVCAR8 cells in adherent (blue) and TIC growth conditions (orange), treated with NCT-505, NCT-506 or Disulfiram. Graphs represent mean and SEM.

The specific ALDH1A1 inhibitor compounds NCT-505 and NCT-506 have been reported to reduce viability of OV90 cells grown in TIC-enriching spheroid growth conditions (20). To examine the effect of the specific inhibitors on the viability of EOC spheroid and adherent cells, cell viability was measured at increasing doses of the NCT-505, -506, or disulfiram. OV90 had the strongest expression of ALDH1A1 by Western blot analysis and showed the highest level of ALDH activity of the EOC cell lines tested (**Figure 1**). In contrast to the effect of disulfiram, the NCT-505 and -506 inhibitors did not achieve the differential effect on viability of OV90 spheroids compared to OV90 cells grown adherently (**Figure 3A**). In OVCAR3 cells, disulfiram and NCT-506 significantly reduced viability of spheroids compared to adherent cells, but NCT-505 did not (**Figure 3B**). Finally, in the low ALDH activity cell line OVCAR8, both NCT-505 and -506 did reduce viability of spheroids compared to adherent (**Figure 3C**), but at a lower potency compared to disulfiram. Disulfiram significantly reduced spheroid viability compared to adherent cell viability in all three cell lines, consistent with previous reports (15) suggesting its broader effects beyond ALDH inhibition and a superior ability to selectively reduce TIC viability.

### Sphere Formation Capacity of Ovarian Cancer Cells is Reduced by ALDH Inhibitors

**Figure 4:**
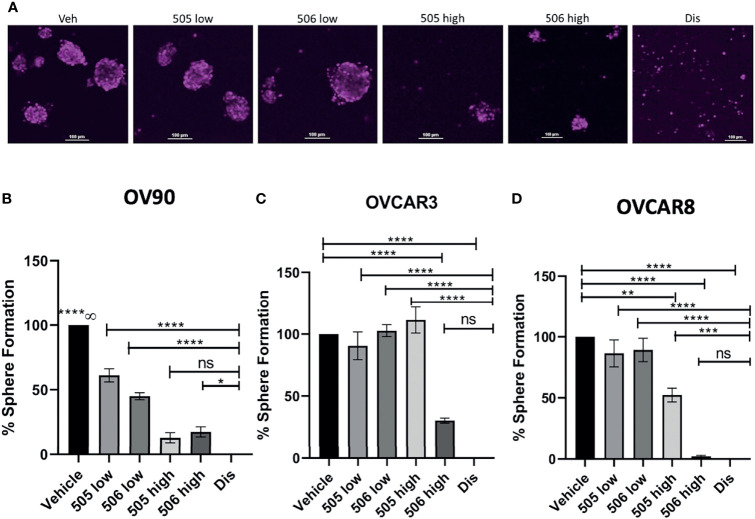
ALDH inhibitor activity on EOC cell sphere formation. **(A)** Representative images of DRAQ5 stained spheres formed by OV90 cells at day 7 after indicated treatments. Scale bar is 100 µm. **(B)** OV90, **(C)** OVCAR3 and **(D)** OVCAR8 cells were plated in TEM in ULA plates at 2,000 cells/well in the presence of the indicated drugs or vehicle (0.1% DMSO). 505 low dose = 2 μM, 505 high dose = 20 μM, 506 low dose = 45 μM, 506 high dose = 100 μM, disulfiram = 250 nM. The cells were maintained in TEM for 7 days, media and drugs were replenished every 48 H and on day 7 images were acquired of spheroids for quantification of number of spheroids measuring >1,000µm^2^. Graphs represent mean and SEM of each treatment, from 3 independent experiments, ns, not significant; ∞, compared to all groups, *p < 0.05, **p < 0.01, ***p < 0.001, ****p < 0.0001, compared to vehicle control.

Sphere formation in EOC cells is important to maintaining TIC survival (21) and inhibition of ALDH by disulfiram has been shown to reduced sphere formation (15). In this study, we also wanted to investigate whether inhibiting ALDH1A1 activity was sufficient to inhibit sphere formation. To test this, cells were grown in the presence of NCT-505, -506 or disulfiram for 7 days and images were acquired to quantify the spheroids that formed (**Figure 4A**). As expected, disulfiram was the most effective in inhibiting sphere formation compared to vehicle control for all lines tested (**Figures 4B–D**). The effect of the ALDH1A1-specific inhibitors on sphere formation was dose-dependent. Using NCT-505 and -506 at the calculated IC_50_ viability dose (**Figure 4**, “high”), sphere formation efficiency was significantly reduced compared to the vehicle control in OV90 and OVCAR8 (**Figures 4B, D**). However, at the dose that was inhibitory to ALDH activity (**Figure 4**, “low”) the NCTs were ineffective in OVCAR3 and OVCAR8, but did reduce sphere formation in OV90, suggesting higher dependence on ALDH1A1 activity in OV90 compared to the other cell lines (**Figures 4B–D**). Interestingly, at the high dose, NCT-506 had a greater effect than NCT-505 in OVCAR3 and OVCAR8, almost equaling the effect of disulfiram (**Figures 4C, D**).

### ALDH Inhibitors Reduce Carboplatin-Treated Viable Cells After Relapse *In Vitro*

**Figure 5:**
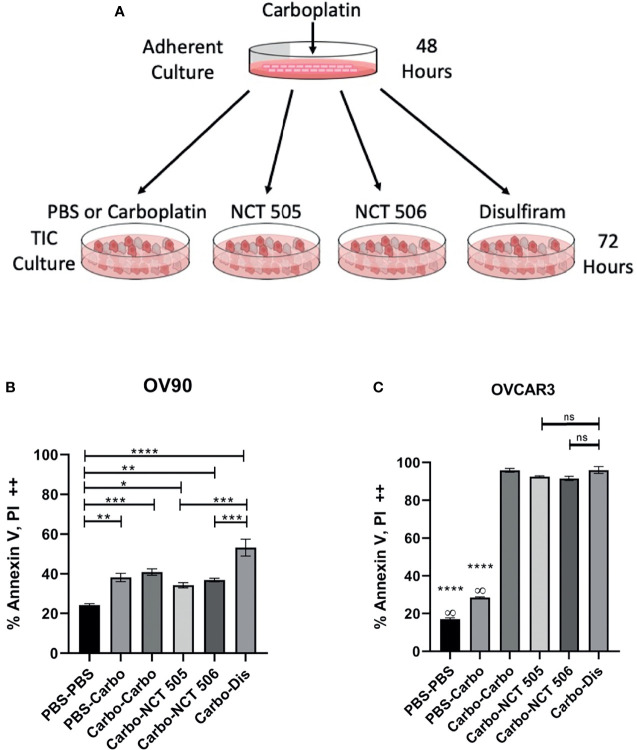
The effect on cell death of ALDH inhibitors in an *in vitro* relapse model. **(A)** Schematic of *in vitro* relapse model outlining 48-hour treatment with carboplatin in adherent cells followed by 72-hour treatment with the NCTs and disulfiram in TIC-enriching conditions. **(B)** OV90 and **(C)** OVCAR3 cells were treated with carboplatin at IC_50_ doses (30 µM and 12.5 µM respectively) for 48 h in adherent conditions, then washed and replated in spheroid conditions for 72 h in the presence of IC_50_ doses of NCT-505, -506, and disulfiram. Cell death was quantified by flow cytometry analysis of cell populations double positive for both cell death markers Annexin-V and PI. Graphs represent mean and SEM of each treatment, from 3 independent experiments, ∞, compared to all groups; ns, not significant, *p < 0.05, **p < 0.01, ***p < 0.001, ****p < 0.0001, compared to vehicle control.

Disulfiram was an attractive candidate from our previous study as it showed better anti-tumor activity towards EOC spheroids than adherent cells, and was able to prevent relapse as a maintenance drug after carboplatin treatment *in vitro* and *in vivo* (15). Therefore we investigated the specific ALDH1A1 inhibitors for their efficacy against spheroid viability after carboplatin treatment, compared to disulfiram in *in vitro* relapse experiments. OV90 and OVCAR3 cells were treated with carboplatin or vehicle for 48 h in adherent growth conditions, then washed and replated in spheroid conditions in the presence of carboplatin, disulfiram, NCT-505 or -506 for 72 h (**Figure 5A**). OV90 cells tend to be more resistant to platinum than OVCAR3 cells. In OV90 spheroids, NCT-505 and -506 after carboplatin increased cell death compared to vehicle-treated spheroids (**Figure 5B**), but did not extend the cell death effect of carboplatin as a secondary treatment. Disulfiram significantly increased cell death compared to vehicle-treated OV90 spheroids, and compared to spheroids treated with NCT-505 and -506 (**Figure 5B**). In OVCAR3 spheroids, there was a significant amount of cell death where carboplatin was given to adherent cells while the secondary treatment given in spheroid growth conditions did not induce additional cell death (**Figure 5C**), as OVCAR3 cells show greater sensitivity to carboplatin than OV90 cells.

### Disulfiram Reduces Classical NFκB Activation in OV90 and OVCAR8 TICs

**Figure 6:**
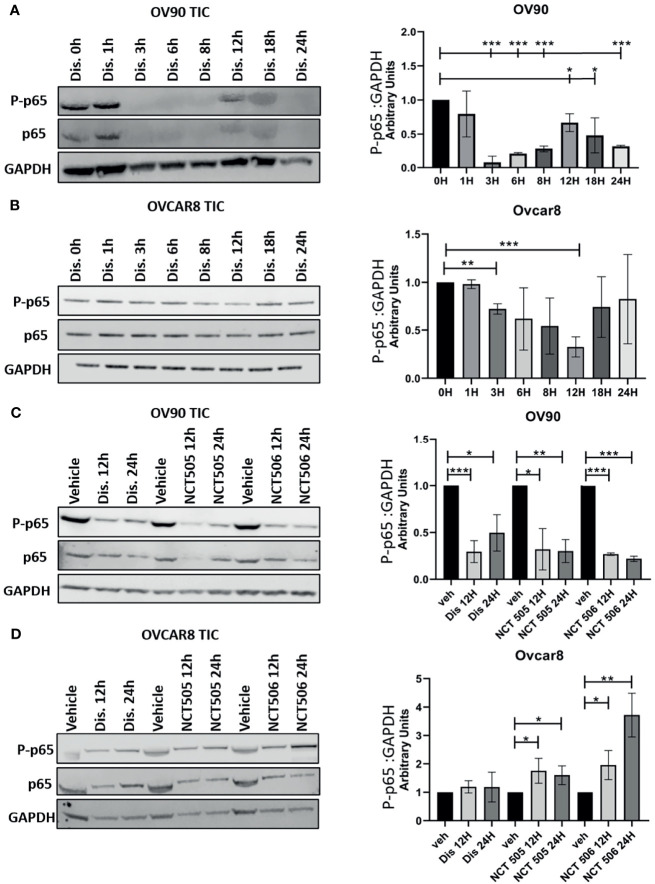
Detection of classical NFκB activation in OV90 and OVCAR8 TICs by Western blot analysis. Following treatment with disulfiram, lysates of **(A)** OV90 and **(B)** OVCAR8 TICs were probed for the expression of phosphorylated p65 at the indicated time points. **(C)** OV90 and **(D)** OVCAR8 TICs were probed for the expression of phosphorylated p65 12 and 24 h after treatment with disulfiram, NCT-505, and NCT-506. Results were quantified comparing ratios of densitometry measurements of phosphorylated p65 to the normalized loading control. The resulting ratios are summarized by the graphs for each indicated cell line and treatment. Graphs represent mean and SEM of each treatment, from 3 independent experiments, *p < 0.05, **p < 0.01, ***p < 0.001, compared to vehicle control.

Classical NFκB signaling is elevated in numerous cancers and has been reported to support TIC proliferation (17, 22–24). Reduced NFκB signaling would be a beneficial component of treatment of ovarian TICs, and ultimately recurrent EOC, therefore we examined the effect of each ALDH inhibitor on NFκB activation. Following treatment of OVCAR8 and OV90 TICs with disulfiram at several time points, we measured the expression of p65, which when phosphorylated, signifies the activation of classical NFκB. In OV90 TICs, we observed expression of phosphorylated p65 at the 0, 1, 12, and 18-hour time points, with significantly reduced expression at hours 12 and 18 h compared to hour 0 (**Figure 6A**). In the OVCAR8 TICs, p65 expression patterns differed from that of OV90 TICs after treatment with disulfiram; p65 was observed at all time points, but was significantly reduced at 3 and 12-hour timepoints (**Figure 6B**). OV90 and OVCAR8 TICs were also treated with NCT-505 and -506 and their effects on phosphorylated p65 expression were compared to those of disulfiram at 12 and 24 h. OV90 TICs showed significantly reduced expression of phosphorylated p65 treated with disulfiram and the NCT-505 and -506 ALDH1A1-specific inhibitors (**Figure 6C**). However, OVCAR8 TICs showed increased expression of phosphorylated p65 at 12 and 24 h compared to vehicle following treatment with both NCT-505 and NCT-506 (**Figure 6D**). This suggests that the effect of disulfiram on TICs to reduce NFκB activation depends on the endogenous level of p65 activation. We examined the effect of disulfiram on OVCAR8 viability with inhibition of classical NFκB signaling, using an IKKβ inhibitor, Inhibitor IV. Using a dose that did not significantly affect the viability of OVCAR8 TICs, but did inhibit p65 phosphorylation (**Supplementary Figures 1A, B**), 0.312 µM of Inhibitor IV was added to the OVCAR8 TICs with disulfiram to examine viability. The effect of disulfiram at its previously demonstrated cytotoxic concentrations was attenuated when coupled with Inhibitor IV (**Supplementary Figure 1C**). This finding shows that exogenous NFκB inhibition eliminates the effect of disulfiram on OVCAR8 TICs and suggests that disulfiram confers its cytotoxic effect specifically by inhibiting classical NFκB *via* the inhibition of p65 phosphorylation.

## Discussion

The prominence of ALDH1A1 in ovarian TICs is well-characterized as the enzyme bolsters chemoresistance and augments drug metabolism (11, 17). In the present study, we aimed to better establish the utility in targeting ALDH1A1 as a method of eradicating TICs and expanding therapy options for the prevention of EOC relapse. By treating OV90, OVCAR3, and OVCAR8 TICs, we compared the effects of the broad ALDH inhibitor disulfiram to that of two ALDH1A1-specific inhibitors, NCT-505 and NCT-506. We found that treatment with the NCTs proved to be less effective against TIC survival than disulfiram, thus detracting from ALDH1A1 being the sole target to consider when treating ovarian TICs, and suggesting that disulfiram exerts its anti-TIC effect by means additional to ALDH inhibition.

Our work builds on the results of a previous study where numerous ALDH1A1 inhibitors were synthesized and tested for biological activity, and analyzed for their efficacy against the viability of EOC lines SKOV-3 and OV90 in different culture conditions (20). In testing for ALDH activity in an expanded set of EOC cell lines, we found that OV90 and OVCAR3 cells had the highest ALDH activity levels, justifying their use in our experiments, and that OVCAR8 had substantially less ALDH activity, which we used as a comparator. We found the activity of NCT-505 and -506 against TICs in all three cell lines to be variable showing that neither NCT selectively targets TICs over adherent cells. We have previously shown that disulfiram significantly diminished cell viability of EOC cell lines in TIC-enriching culture conditions (15) which we reproduced in the current study. Additionally, compared to the NCTs, disulfiram potently reduced sphere formation in all three tested cell lines. The ability of ovarian cancer spheres to form has been shown to be affected by modulations in microRNA, the Wnt-signaling pathway, and treatment with xenohormones (25–27). It remains unclear whether treatment with disulfiram affects these processes related to sphere formation. However, given its ability to completely disable sphere formation, disulfiram is more likely than the NCTs to have a connection to the reported regulatory mechanisms of sphere formation.

In the context of cell death, disulfiram outperformed the two strongest inhibitor analogs from Yang et al. (20) with a greater potency than both compounds, which was the first indication that ALDH1A1 inhibition alone may not be the optimal way to kill TICs and prevent relapse. Whether it be a result of ALDH inhibition or the induction of certain cell death pathways, the specific mechanism by which disulfiram causes cell death has yet to be fully elucidated. It has been reported that the metabolites of disulfiram cause its cytotoxic effects irrespective of its targeting of ALDH (28). Disulfiram has been shown to be associated with a variety of cell death pathways, namely, necroptosis, autophagy, ferroptosis, and apoptosis (29–31) highlighting the broad extent of its cellular effects. Further investigation into the specific effects that disulfiram has on cell death is needed and will point to additional therapeutic targets in ovarian TICs and help clarify the mechanism of action of disulfiram.

Increased activation of classical NFκB has been observed in the TICs of several cancers and is associated with cell adhesion, proliferation, and cell survival (17, 22–24) making its inhibition desirable. After treatment with disulfiram at several time points, we probed OV90 and OVCAR8 TICs for the expression of phosphorylated p65, which indicates classical NFκB activation and subsequent cell survival and proliferation (17, 32). We found reduced NFκB activation after disulfiram treatment in the high-ALDH-activity OV90s compared to the low-ALDH-activity OVCAR8s. We observed fluctuations in p65 expression over a 24-hour period in both OV90 and OVCAR8 TICs after treatment with disulfiram. A dose-dependent reduction in NFκB caused by disulfiram has been previously reported (33). Our findings indicate that reduction of NFκB activity caused by disulfiram has temporal variation in addition to its dependency on dose. Importantly, disulfiram reduced NFκB activation at a considerably lower dose compared to the NCTs giving it a compelling advantage over the NCTs as a therapeutic option for the prevention of recurrence in EOC. Our findings show NCT-505 and -506 reduced phosphorylated p65 expression after 12 and 24 h in OV90, but not OVCAR8 cells, but at a substantially lower potency compared to that of disulfiram. Using Inhibitor IV at a sub-lethal dose in combination with disulfiram, we demonstrated that the efficacy of disulfiram in reducing TIC viability was eliminated, thus confirming the anti-TIC activity of disulfiram was *via* inhibition of p65 phosphorylation in classical NFκB signaling.

We have previously demonstrated that disulfiram successfully prolongs survival in an *in vivo* relapse model (15) and we compared the efficacy of the NCTs and disulfiram in killing TICs in an *in vitro* relapse model. OVCAR3 cells are sensitive to platinum-based therapies (34) which explain the considerable amounts of cell death we observed across all treatments in OVCAR3 TICs. As a second line treatment in OV90s, disulfiram caused significantly more cell death than both NCT-505 and NCT-506. The NCTs as a second line treatment induced similar death levels as carboplatin, which is typically ineffective as recurrent ovarian cancer patients often acquire platinum-resistance (35). This result highlights the versatility of disulfiram in both inhibiting ALDH and enhancing cell death in a relapse model. A previous finding with NCT-501, an analog from which NCT-505 and -506 were derived, demonstrated that it was ineffective in reducing tumor size *in vivo* on its own (36). Our inconsistent findings using the NCTs *in vitro* made the compounds unsuitable options for *in vivo* experiments in this study.

In comparing disulfiram to NCT-505 and -506, it is evident that disulfiram is superior to the specific inhibitors of ALDH1A1 in targeting EOC TICs and treating relapse. We have demonstrated that, in addition to inhibiting ALDH, disulfiram reliably reduced TIC viability and induced the most cell death in a platinum-resistant relapse model. Unlike the NCTs, disulfiram effectively restricted sphere formation and it reduced NFκB activation with greater potency than the NCTs, further enhancing its cytotoxicity to TICs. Further elucidation of the mechanism of disulfiram is necessary and will lead to a refined understanding of the best way to eliminate TICs and make future treatment of recurrent EOC more durable. While inhibition of ALDH should not be ignored pharmacologically, therapies for recurrent EOC would greatly benefit from the development of drugs with additional action similar to disulfiram. Drugs like disulfiram with useful, off-target cellular effects in TICs would be more advantageous in preventing relapse compared to the use of specific compounds that inhibit a single isozyme of ALDH.

## Materials and Methods

### Antibodies and Reagents

Carboplatin was purchased from the Tocris Bioscience (Minneapolis, MN) (cat. No. 2626) and dissolved in phosphate buffered saline (PBS). ALDH1A1 inhibitors NCT 505 (NCGC00384406) and NCT 506 (NCGC00386123) were synthesized as described (20) and was provided by Dr. Shyh-Ming Yang (National Center for Advancing Translational Sciences). Propidium Iodide (PI) was from Roche (Cambridge, MA) and Annexin V-FITC (556420) was from BD Biosciences (San Jose, CA). IKKβ inhibitor (Inhibitor IV, 401484) was from Millipore Sigma (Burlington, MA). ALDH1A1 (ab52492) and ALDH1A2 (ab96060) antibodies were from Abcam (Cambridge, MA). GAPDH (MAB374) was from Millipore Sigma (Burlington, MA). Phosphorylated p65 (3033L) cleaved poly (ADP-ribose) polymerase (9541) were from Cell Signaling Technologies (Danvers, MA). Total p65 (10815) was from Santa Cruz Biotechnologies (Dallas, TX).

### Cell Lines and Culture Conditions

Ovarian cancer lines OV90, OVCAR3, and OVCAR8 were obtained from the American Type Culture Collection (ATCC, Manassas, VA). All cultures were maintained at 37°C in 5% CO_2_. Cell lines were cultured in RPMI (Thermo Fisher Scientific, Waltham, MA) medium containing 10% (v/v) fetal calf serum (FCS), penicillin (100 units per ml) and streptomycin (100 units per ml). TIC-enriching culture medium (TEM) was previously described (17). TIC-enriching spheroid culture conditions were generated by maintaining cells in ultra-low attachment (ULA) plates or flasks (Corning, NY). Experiments involving the TIC-enriched spheroid populations were grown for 3 days in defined medium in ULA plates before drug treatments were performed.

### Western Blot Analysis

OV90 and OVCAR8 cells were grown in as spheroids for 3 days in TEM, and treated with disulfiram and the NCTs as indicated. Whole cell lysates were collected at the indicated time points post treatment, by pelleting spheroids and removing growth medium and washing in PBS. Lysates were collected in lysis buffer: RIPA buffer (Thermo Scientific, Waltham, MA) containing 1× protease inhibitor (Halt, Thermo Fisher Scientific, Waltham, MA) 1× phosphatase inhibitor (Phos-STOP, Millipore Sigma, Burlington, MA). After a brief incubation on ice, the lysates were homogenized by passing the samples through 26-G needles for 5 strokes, followed by centrifugation at 16,000*g*, 4°C for 20 min to collect the supernatant. Protein concentration was quantified by microbicinchoninic acid (BCA) assay (Thermo Fisher Scientific, Waltham, MA). Lysates (40 µg) were separated by SDS-PAGE under reducing conditions, transferred onto PVDF membranes, and blocked in 5% non-fat milk in Tris-buffered saline containing 0.1% Tween 20 (TBST). Membranes were incubated with primary antibodies diluted in 1% non-fat milk in TBST overnight at 4°C, washed with TBST, and then incubated with secondary HRP-conjugated mouse or rabbit IgG as appropriate. Images were generated using the Odyssey system and software (LI-COR Biosciences, Lincoln, NE).

### Cell Viability

Cell viability was assessed as previously described (15) using CellTiter-Glo (Promega, Madison, WI, USA) according to the instructions of the manufacturer.

### Sphere Formation Efficiency

Sphere formation of OV90, OVCAR8 and OVCAR3 cells was performed as previously described (15). Cells were seeded at 2,000 cells/well in 96-well ULA plates (3474, Corning, Corning, NY), in TEM with indicated drugs for 7 days, fresh culture medium containing growth factors and the drugs was replenished every 48 h. After 7 days the spheres were incubated with DRAQ5 (Thermo Fisher Scientific, Waltham, MA, USA) at 1 µM for 15 min prior to imaging using an inverted Nikon Ti2-E microscope (Nikon, Melville, NY), equipped with a Yokogawa SoRa CSU-W1 spinning disk unit (Yokogawa, Sugar Land, TX) and a BSI sCMOS camera (Teledyne Photometrics, Tuscon, AZ). Images of spheroids were acquired using the automated acquisition module (JOBS) to image each well using a 10× plan-apochromat N.A. 0.45 objective lens, 200 ms exposure. Quantification of spheroids was performed using NIS Elements software version 5.30 (Nikon, Melville, NY). Images were processed using Segment.ai trained on human-recognized, hand-traced spheroids. Segment.ai learned how to trace spheroids in subsequent images. The number of spheroids measuring an area of >1,000 μm^2^ were counted.

### Flow Cytometry Assays for ALDH Activity and Cell Death

ALDH enzymatic activity was quantified using the ALDEFLUOR kit from Stem Cell Technologies (Seattle, WA), used according to the instructions of the manufacturer and as previously described (15). Briefly, cells were plated in spheroid culture conditions for 72 h prior to drug treatment with NCT-505 (11.45 μM), NCT-506 (103 μM), and disulfiram (250 nM). Following 72 h of drug treatment, 1.0 × 10^5^ viable cells were incubated with ALDEFLUOR assay buffer containing the active substrate for 60 min at 37°C or were incubated with an ALDH inhibitor diethylaminobenzaldehyde (DEAB) to serve as a negative control in tandem. Cell death was measured from the *in vitro* relapse assay, as described previously (15). For the *in vitro* relapse model, cells were grown in adherent conditions in the presence of carboplatin or PBS control for 48 h, then trypsinized, and 1.0 × 10^5^ cells re-plated in fresh TIC-enriching media in ULA plates and treated with the indicated drugs for 72 h. The cells were collected and a total count was performed, and 1.0 × 10^5^ cells from each treatment were stained with Annexin V-FITC according to the protocol of the manufacturer. After the final wash step, PI was added at 1:10 dilution in PBS and incubated for 15 min, protected from light and directly after was analyzed. Fluorescence was detected on a flow cytometer, and analyzed using FlowJo software (Becton, Dickinson and Company, NJ).

### Statistical Analysis

*In vitro* assays were performed in triplicate on three independent occasions and were analyzed with t-tests or one-way ANOVA with post-tests where applicable. Results are presented as mean ± SEM with p-values ≤0.05 considered significant. Statistical analyses were performed using Prism 8.0 software (GraphPad, San Diego, CA, USA).

## Data Availability Statement

The original contributions presented in the study are included in the article/**Supplementary Material**. Further inquiries can be directed to the corresponding author.

## Author Contributions

The study was conceptualized by BSH and CMA. Methodology for quantifying spheroid formation was designed by MJK. Data curation and formal analysis was performed by MWC, RDK, and BSH. Original draft was written by MWC and BSH. All authors listed have made a substantial, direct, and intellectual contribution to the work and approved it for publication.

## Funding

Funding was provided by the Intramural Research Program, Center for Cancer Research, National Cancer Institute (ZIA BC011054).

## Conflict of Interest

The authors declare that the research was conducted in the absence of any commercial or financial relationships that could be construed as a potential conflict of interest.

## Publisher’s Note

All claims expressed in this article are solely those of the authors and do not necessarily represent those of their affiliated organizations, or those of the publisher, the editors and the reviewers. Any product that may be evaluated in this article, or claim that may be made by its manufacturer, is not guaranteed or endorsed by the publisher.

## Supplementary Material

Supplementary Figure 1 OVCAR8 cells grown in TIC-enriching conditions treated with Inhibitor IV, were tested for **(A)** effects on viability and **(B)** inhibition of phosphorylation of p65. **(C)** Viability of OVCAR8 cells in TIC growth conditions was assessed following combinatorial treatment with Inhibitor IV and disulfiram (green) and disulfiram and vehicle (orange). Graphs represent mean and SEM.
