# Supplementary figures and images for: Disulfiram Transcends ALDH Inhibitory Activity When Targeting Ovarian Cancer Tumor-Initiating Cells
Source: Front Oncol. 2022 Mar 17;12:762820. doi: 10.3389/fonc.2022.762820 (PMC8967967; doi:10.3389/fonc.2022.762820)

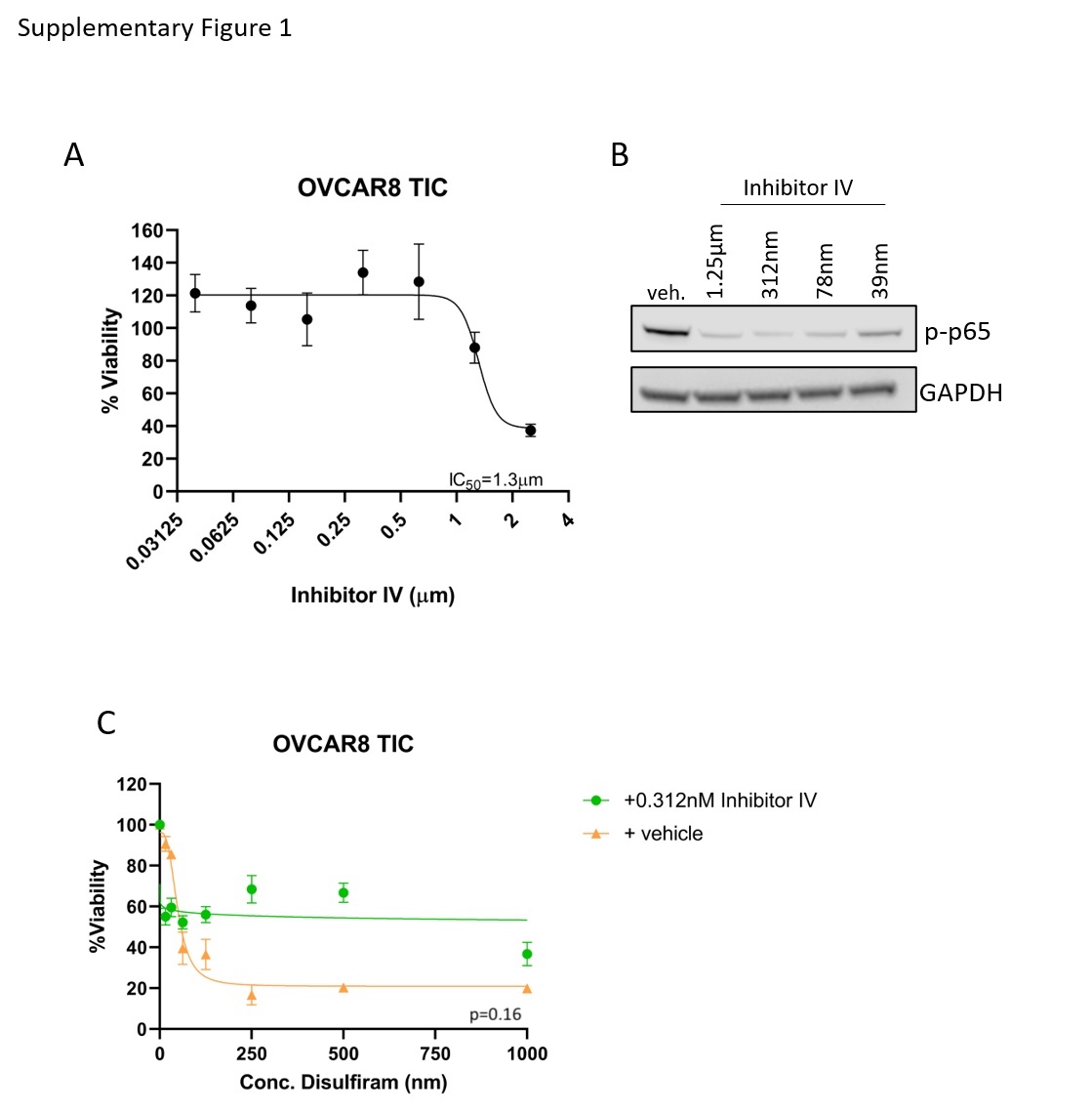

Supplement: Supplementary Figure 1 — OVCAR8 cells grown in TIC-enriching conditions treated with Inhibitor IV, were tested for (A) effects on viability and (B) inhibition of phosphorylation of p65. (C) Viability of OVCAR8 cells in TIC growth conditions was assessed following combinatorial treatment with Inhibitor IV and disulfiram (green) and disulfiram and vehicle (orange). Graphs represent mean and SEM. [file Image_1.jpeg]
